# Acute Myeloid Leukemia: From Biology to Clinical Practices Through Development and Pre-Clinical Therapeutics

**DOI:** 10.3389/fonc.2020.599933

**Published:** 2020-12-09

**Authors:** Xavier Roussel, Etienne Daguindau, Ana Berceanu, Yohan Desbrosses, Walid Warda, Mathieu Neto da Rocha, Rim Trad, Eric Deconinck, Marina Deschamps, Christophe Ferrand

**Affiliations:** ^1^ Inserm EFS BFC, UMR1098 RIGHT, University Bourgogne Franche-Comté, Besançon, France; ^2^ Department of Hematology, University Hospital of Besançon, Besançon, France

**Keywords:** acute myeloid leukemia, immunotherapies, CAR T cells, management, clinical trials

## Abstract

Recent studies have provided several insights into acute myeloid leukemia. Studies based on molecular biology have identified eight functional mutations involved in leukemogenesis, including driver and passenger mutations. Insight into Leukemia stem cells (LSCs) and assessment of cell surface markers have enabled characterization of LSCs from hematopoietic stem and progenitor cells. Clonal evolution has been described as having an effect similar to that of microenvironment alterations. Such biological findings have enabled the development of new targeted drugs, including drug inhibitors and monoclonal antibodies with blockage functions. Some recently approved targeted drugs have resulted in new therapeutic strategies that enhance standard intensive chemotherapy regimens as well as supportive care regimens. Besides the progress made in adoptive immunotherapy, since allogenic hematopoietic stem cell transplantation enabled the development of new T-cell transfer therapies, such as chimeric antigen receptor T-cell and transgenic TCR T-cell engineering, new promising strategies that are investigated.

## Introduction

In 2010 and 2017, the European LeukemiaNet (ELN) international expert panel published recommendation for the diagnosis and management of acute myeloid leukemia (AML) (1, 2). Furthermore an update of the WHO classification in 2016 provided a few changes to the previously described disease categories (3). This new classification of AML is focused on the determination of genetic abnormalities *via* molecular based investigations, in addition to cytogenetic abnormalities for *de novo* AML, in contrast to myelodysplasia- and therapy-related AML (MRC-AML and tAML). Moreover, genetic and molecular characterizations of AML resulted in the establishment of the 2017 ELN risk stratification (2). This article reviews current AML pathogeneses and novel therapies.

## Biology and Pathogenesis

### Leukemogenesis of AML Results From Cytogenetic and Genetic Abnormalities

During the last decade, some progress has been made towards a better understanding of AML disease pathogenesis (4). The Cancer Genome Atlas Research Network has described eight functional categories of genes that are commonly mutated in *de novo* AML (5): signaling genes (FLT3, KRAS, NRAS and KIT mutations); epigenetic homeostasis genes with 2 subcategories, chromatin-modifying genes (ASXL1 and EZH2 mutations, MLL fusions) and methylation-related genes (DNMT3A, TET2, IDH1, and IDH2 mutations); nucleophosmin gene (NPM1 mutations); spliceosome-complex genes (SRSF2, SF3B1, U2AF1, and ZRSR2 mutations); cohesin-complex genes (RAD21, STAG1, STAG2, SMC1A, SMC3 mutations), myeloid transcription factors (RUNX1, CEBPA, and GATA2 mutations, RUNX1-RUNX1T1, PML-RARA, MYH1-CBFB fusions); and tumor suppressive genes (WT1, TP53 mutations with PTEN and DMM2 deregulations); (**Table 1**) (4, 6). Two or more of these driver mutations have been identified in 86% of the patients. Combinations of these driver mutations may be compartmentalized into 11 classes with different overall survival rates (7). Thus, two new provisional entities (AML with mutated RUNX1 and AML with BCR-ABL1) have been included in the update of the WHO classification (3) and mutations in three genes (RUNX1, ASXL1 and TP53) have been added to the risk stratification of the 2017 ELN recommendation (2), which could guide new therapies (8). These mutations have been confirmed in the largest mutational study conducted thus far, the Beat AML cohort, with similar frequency of mutations (9).

**Table 1:** Eight functional categories of genes mutations in acute myeloid leukemia (AML).

Functional categories		Genes involved	Frequency
Signaling genes		FLT3, KRAS, NRAS, and KIT mutations	59%
Epigenetic homeostasis genes	Chromatin-modifying genes	ASXL1 and EZH2 mutations, MLL fusions	30%
	Methylation-related genes	DNMT3A, TET2, IDH1, and IDH2 mutations	44%
Nucleophosmin gene		NPM1 mutations	27%
Spliceosome-complex genes		SRSF2, SF3B1, U2AF1, and ZRSR2 mutations	14%
Cohesin-complex genes		RAD21, STAG1, STAG2, SMC1A, SMC3 mutations	13%
Myeloid transcription factors		RUNX1, CEBPA, GATA2 mutations	22%
		RUNX1-RUNX1T1, PML-RARA, MYH11-CBFB fusions	18%
Tumor suppressive genes		WT1, TP53 and PHF6 mutations (PTEN and DMM2 deregulations)	16%

Functional categories of genes mutations in AML. Adapted from The Cancer Genome Atlas (TCGA). FLT3, Fms-Like Tyrosine Kinase 3. KRAS, KRAS Proto-Oncogene. NRAS, NRAS proto-oncogene. KIT, c-KIT proto-oncogene. ASXL1, Additional Sex Combs Like 1. EZH2, Enhancer of Zeste Homolog 2. MLL, Mixed Lineage Leukemia. DNMT3A, DNA Methyl-Transferase 3 alpha. TET2, Ten-Eleven Translocation Methylcytosine Dioxygenase 2. IDH1/2, Isocitrate Dehydrogenase 1 and 2. NPM1, Nucleophosmin 1. SRSF2, Serine and arginine Rich Splicing Factor 2. SF3B1, Splicing Factor 3B Subunit 1. U2AF1, U2 small nuclear RNA Auxiliary Factor 1. ZRSR2, Zinc finger CCCH-type, RNA binding motif and Serine/arginine Rich 2. RAD21, RAD21 cohesin complex component. STAG1/2, Stromal Antigen 1 and 2. SMC1A, Structural Maintenance of Chromosomes 1A. SMC3, Structural Maintenance of Chromosomes 3. RUNX1, Runt-related transcription factor 1. RUNX1T1, RUNX1 partner Transcriptional co-repressor 1. CEBPA/B, CCAAT/Enhancer-Binding Protein alpha and beta. GATA2, GATA-binding protein 2. PML-RARA, promyelocytic leukemia/retinoic acid receptor alpha. MYH11, Myosin Heavy chain 11. WT1, Wilms’ Tumor 1. PTEN, Phosphatase and Tensin homologue. DMM2, Double Mouse Minute 2 homologous. PHF6, Plant Homeodomain-like Finger protein 6.

Most cases of AML present with clonal heterogeneity at the time of diagnosis (5, 8). Driver mutations, such as DNMT3A, TET2, and ASXL1, appear early in AML clones (5) and in the myelodysplastic syndrome (MDS) (10). Nevertheless, these 3 mutations are also found in healthy donors, and more frequently in elderly individuals along with clonal hematopoiesis of indeterminate potential (CHIP) and age-related clonal hematopoiesis (ARCH) (11). Most mutations found in AML genomes occur randomly in hematopoietic stem and progenitor cells (HSPC) that result in clonal evolution. These mutations preexist in the background before HSPC acquire the initiating mutations (NPM1, DNMT3A or IDH1) leading to AML pathogenesis (12). Only a small number of genic mutations are required for AML pathogenesis, and one or two additional cooperative mutations are needed to generate the founding malignant clone. The most frequent associations are NPM1+DNMT3A, NPM1+IDH1, NPM1+FLT3, DNMT3A+IDH1, and DNMT3A+FLT3. Similarly, clonal evolution is also detected when relapsing (13).

During the course of treatment, AML cells acquire a small number of additional cooperating mutations from the primary clone that contribute to disease progression or relapse (12). Selection of a dominant clone and/or additional mutations may be caused by inadequate treatment, such as the use of a drug despite apparent drug specific resistance or treatment that is not sufficiently intensive (13). Patients who are unable to tolerate aggressive consolidation have poorer outcomes (2). Specific additional mutations may result in resistance to chemotherapy and may play an important role in relapses. Mutational analyses of samples of primary and relapsing tumors indicate that chemotherapy may induce a substantial effect on the mutation spectrum at relapse, probably *via* DNA damage (13). Nevertheless, these same results suggest that mutations are neither associated with generalized genomic instability (13), nor with recurrent cohesin complex gene mutations (12). Clonal evolution may be caused by a certain type of therapy itself (13). Therefore, targeted therapies may be used in order to reduce the side effects of mutagenesis, while avoiding the use of cytotoxic drugs. Thus, continuous AML genome evolution in an individual patient would find and eradicate all subclones. Although only a tiny fraction of the total mutations are relevant for pathogenesis, some mutated non-genic regions are also described, suggesting functional properties that need further investigation (12). Lastly circular RNA profiling has been performed in cytogenetically normal AML as a proof-of-principle and has allowed 3 clusters with clinical and functional significances to be characterized (14). High levels of KLHL8 and FCHO2 circular RNA are known to be associated with better outcomes.

Recently, AML pathogenesis has been modeled by expression of distinct leukemia-associated mutations (15). “TYPE-A mutations” (expression of AML-associated fusion genes such as MLL, CBF or RARA fusions) are necessary to maintain transformed phenotypes. “TYPE-B mutations” (constitutively activated kinases by fusion or mutation such as ABL, PDGFR, KIT, FLT3, JAK2, or signaling mediators activating the RAS-MAPK pathway) lead to the development of a lethal myeloproliferative disorder. “TYPE-C mutations” (characterizing clonal hematopoiesis and preleukemic states including point mutations in IDH1/2, DNMT3A, TET2, NPM1c) which are referred to as “seed mutations,” based on their potential. Coexpression of TYPE-A and TYPE-B mutations cooperates to induce AML-like phenotype following a short latency, whereas TYPE-C mutation collaborate with TYPE-A and TYPE-B mutations resulting in AML with high penetrance in mice. Targeting of TYPE-A mutations has been reported as the best path to take in order to cure respective potent driver oncogenes. Although targeting of TYPE-B mutations may be insufficient to eliminate the disease, it may be combined with conventional chemotherapy. Finally, targeting of TYPE-C mutations, such as mutant IDH1/IDH2, has recently demonstrated potential as a promising antileukemic therapeutic strategy (16–19).

### Leukemic Stem Cells Develop From Cell-of-Origin of Leukemia Through a Number of Transforming Events

Studies based on mouse models have highlighted that AML, driven by potent oncogenes, such MLL fusion, may have developed *via* committed myeloid progenitors (CMP) whereas AML without any major cytogenetic abnormalities may emerge due to a combination of preleukemic initiating events arising in the hematopoietic stem cell (HSC) pool (15). Strong oncogenes mostly originate in CMPs, following which a few cooperating mutations may enable development of AML. In a minor fraction, it may originate in the HSC compartment leading to a particularly invasive and highly resistant phenotype. A high fraction of AML may be produced by the early cooperation of multiple mutations in the HSC compartment, which provides a clonal advantage leading to a preleukemic state, after which the gain of additional mutation may lead to the development of symptomatic AML. Thus, many AML samples show evidence of cellular origin associated with a hierarchical organization model of leukemia, driven by a small population of stem cells, leukemia-initiating cells (LIC) or leukemic stem cells (LSC). These cells are capable of self-renewal in serial mice transplantations and partially differentiate into non-LSC bulk blasts that resemble the original disease, although non-LSC blasts are unable to self-renew (20). LSC properties are self-renewal, relative quiescence, resistance to apoptosis, and increased drug efflux (21). Most cases of AML in mice and humans originate from a continuum of early multipotent to more differentiated hematopoietic progenitor cells, but about 10-20% of human AML may arise from more immature cells, the long-term HSCs (LT-HSCs) (15). The transforming capacity of particularly potent leukemogenic oncogenes may be influenced by the cell of origin. The role of heterogeneity in AML links clonal evolution and LSC (22).

Xenotransplantation of human AML samples, mostly AML LSCs, in NOD/SCID-gamma null (NSG) mice have highlighted that 80% of CD34^+^ AML cases, which are defined by the presence of CD34 expression on more than 10% of blasts analyzed, contained 2 expanded cell populations with an immune phenotype of CD38^—^CD90^—^CD45RA^+^ or CD38^+^CD110^+^CD123^+^CD45RA^+^ both resembling normal, early and more mature hematopoietic progenitor cells (HPCs) rather than HSCs (23). These two cell populations display leukemia initiating potential upon serial transplantation and are hierarchically ordered; CD38^—^ cells with a higher number of LSCs resembling normal lymphoid-primed multipotent progenitors (LMPP-like LSC) give rise to leukemic granulocyte-macrophage progenitors (GMP-like LSC), but not vice versa, while CD38^+^ cells present less LSC. This suggests that the majority of CD34^+^ AML cells arise from HPCs that have acquired self-renewal properties, rather than from those with a direct HSC origin (15, 20). Despite many lymphoid and myeloid antigens aberrantly expressed in AML (CD7 or CD11b) and complex leukemia-associated phenotype shifts at the time of relapse (24), a number of cell surface markers that are upregulated on CD34^+^CD38^—^LSC compared with normal CD34^+^CD38^—^ HSPC (20) have been identified as follows: CD123 (IL-3 Receptor α chain) (25); CD44 (cell surface glycoprotein ligand for L-selectin) (26); CD47 (ligand for signal regulatory protein alpha, SIRPα) (27); TIM-3 (T cell immunoglobulin mucin-3) (28); CD96 (recognizing the adhesion molecule nectin-like protein-5, necl-5) (29); CD99 (30), CD371 as known as CLL-1 (C-type lectin-like molecule-1) (31); CD32 (Fcγ Receptor II), CD25 (IL-2 receptor); IL1RAP (IL-1 receptor accessory protein) (32, 33); GPR56 (G protein-coupled receptor 56) (34); and CD93 (C-type lectin) (35). However, their stability is unknown because most have not been studied during relapse (20) and only few have been targeted (36, 37). However concerning the CD123 cell surface maker, at relapse CD123^+^CD34^+^CD38^—^ cells were found to increase (20). In addition, CD123 co-segregates with FLT3-internal tandem duplication (ITD) mutation-positive CD34^+^CD38^—^ cells suggesting that CD123 is a robust LSC marker in FLT3-ITD-mutated AML (38). Notably, nearly all human AML CD34^+^ and CD34^—^ cells are CD33^+^ in xenotransplantation (23). On the other hand, above 25% of AML blasts do not express CD34, defined as present on less than 10% of blasts, but are enriched for NPM1 mutation. These are composed of more than 98% of CD34^—^ cells with multiple and nonhierarchically arranged CD34^—^ and CD34^+^ LSC-containing populations (39). Both CD34^—^ and CD34^+^ LSCs represent the same cells with aberrant plasticity of CD34 expression (20, 39). CD34^—^ LSC global transcription profiles seem very similar to those of normal CD34^—^ GMP. However, unlike mature precursors, they express multiple normal stem cell transcriptional regulators that are implicated in LSC functions. This suggests that the nature of the genetic/epigenetic driver events determines a disordered transcriptional program that results in LSCs with differentiation arrested at either the progenitor or precursor stages of hematopoiesis (15, 20, 39). However, clonal composition changes in the majority of AML xenografts in mice, but could also enable delineation of clonal hierarchies and unmask previously undetectable clones (40). New tools such as single-cell sequencing may permit a better understanding of LSC hierarchies and functions (41), and help define specific targeting strategies (42).

Single-cell gene expression preceded by genome-wide transcriptional analysis of AML and chronic myeloid leukemia (CML) LSCs have highlighted several cell markers, such as IL1RAP and CD25 that are overexpressed (42–44). Nevertheless, these AML and CML LSC cell surface makers are also expressed on normal HSCs (45). Interestingly, IL1RAP is described to potentiate multiple oncogenic pathways in AML (46). Targeting the extracellular portion of IL1RAP with monoclonal antibodies, IL1RAP down regulation with short hairpin RNA, and knocking out of *Il1rap via* genetic deletion inhibits AML growth intrinsically, through the induction of differentiation and apoptosis without affecting healthy hematopoietic cells that present a low expression of IL1RAP, and inhibit AML pathogenesis *in vivo*. Inhibition of the canonical IL-1 receptor signaling pathway induces the abolition of IRAK1 expression, which reduces MDS and AML leukemic colony formation and prolongs the survival of xenograft models (47). Indeed the IL-1 receptor complex, formed by IL-1 receptor, IL1RAP, MyD88, IL-1R associated kinases 2 (IRAK2), and IRAK4 (48), induces the activation of IRAK1 and TRAF6, leading to the activation of the IKK complex, which in turn results in activation of NFκB target genes, as well as JNK and p38 (49). This pathway has been described in several cancers and its targeting in AML seems promising (32, 49). Of note, an active super enhancer has been reported with IL1RAP in AML, in samples that also presented FLT3, NPM1, and IDH1 associated mutations (50). In addition to the inhibition of the IL-1 receptor pathway, targeting IL1RAP may also inhibit signaling and AML cell growth occurring *via* the FLT3 and c-KIT pathways with a reduced response to FLT3 ligand, stem cell factor (SCF) and IL-1β (46). Nevertheless, FLT3 mutations independently induce a constitutive activation of the IL1RAP associated FLT3 pathway, but it has been demonstrated that IL1RAP physically interacts with FTL3 and c-KIT, even although FLT3 is mutated. However IL1RAP overexpression occurs early in LSC pathogenesis (43) whereas FLT3 and c-KIT activation mutations seem to be a relatively late event in LSC transformation (51, 52). Furthermore, chronic inflammation due to exposure to IL-1 impairs blood hemostasis and restricts HSC lineage output (53). Continuous exposure to IL-1, in association with IL-6, TNF (tumor necrosis factor) and IFNs (interferons), may promote genomic instability and induce a pre-leukemic stage *via* continuous proliferation, bone marrow niche dysfunction and exposure to reactive oxygen species (ROS), that drive myeloid malignancies such AML (54). IL1RAP is described as an inflammatory regulator and its overexpression in AML may be linked to a proinflammatory state (55).

### Bone Marrow Microenvironment Sustains Blast Proliferation, and Promotes Resistance to Treatment

HSCs in the bone marrow interact with mesenchymal stem/stromal cells (MSC), sinusoidal endothelial cells, osteoblasts, osteoclasts, macrophages, and immune cells, adipocytes, autonomic neurons, extracellular vesicles, extracellular matrix, and soluble factors, including cytokines and growth factors (56). In AML the interaction among blasts, stromal cells and immune cells of the bone marrow (BM) microenvironment, promoted by soluble niche factors, create a niche that sustains blast proliferation and confers chemoresistance by remodeling the BM niche *via* alteration of each cellular constituent (57–59). AML cells induce osteogenic differentiation but inhibit adipogenic differentiation of MSC leukemia growth, whereas normal hematopoiesis is favored by AML-secreted exosomes and pro-inflammatory IL-1, which promote the expansion of AML progenitors and disease progression by activating the IL-1/p38MAPK pathway. Chronic increase of IL-1β with IL1RAP binding expands malignant cells while HSCs are intrinsically depleted (33, 53). Indeed, myeloid differentiation of *Dnmt3a*-mutant LT-HSC seems to be promoted by HSC-extrinsic alterations in aged BM microenvironments, *via* TNFα and Macrophage Colony-Stimulating Factor (M-CSF), that overcome impaired differentiation (60).

Interestingly, IL-1β appears to play a key role in cardiovascular diseases of young patients with CHIP (11). Similarly, overproduction of IL-1β by mutant progenitors damages Schwann cells, leading to neuropathy, increases MSC apoptosis and decreases HSC adhesion molecule expression (43, 59). Cytokines and particularly IL-33, a ligand of IL1RAP, released by AML cause the remodeling of vasculature, reduces its ability to support normal hematopoiesis, and compromise vascular integrity by niche cell based stimulation of myeloid cytokine production. Then FoxO1 and β-catenin stimulate osteoblasts that trigger aberrant Notch signaling in HSCs, and induce leukemic transformation (58). In proteomic and gene expression profiling, the cytokine/chemokine signaling network causes the most striking AML-associated proteomic alteration of BM microenvironment (58). Pro-inflammatory IL-8 is the key central molecule of this network in AML that may show potential as an attractive therapeutic target. MPIF-1 (CCL23), an inhibitory cytokine that inhibits proliferation and differentiation of myeloid precursor cells, is also upregulated. Increases in the network of proteins that regulate osteoblastic and osteoclastic activities have been highlighted in AML BM. Deregulated expression of CXCL12/CXCR4 by stromal cells may impinge on the ability of normal HSCs to find and reside in their quiescence maintaining niches, and promote exhaustion and progressive malignant clonal dominance of these HSCs (59). Contact-dependent transfer of functional mitochondria from MSCs to AML cells has been demonstrated (61). Such mitochondrial transfer is increased under chemotherapy conditions and may lead to resistance to chemotherapy. Similarly, LSCs may highjack the lipolytic role of adipocytes *via* the fatty acid transporter CD36 (62). Interestingly, CD36 is associated with a poor prognosis (63).

Additionally, certain processes that indicate immune dysregulation and leukemia microenvironment remodeling have been described in AML as follows: low neoantigen burden and defective antigen presentation; higher regulatory/suppressive T-cells (Treg) proportion and lower T effector (Teff) proportion; T-cells exhaustion due to upregulation of immune checkpoint ligands and receptors; chronic inflammation and increase of inflammatory macrophage population (M1); increase of myeloid derived suppressor cell population (MDSC) and suppressive macrophage population (M2) derived from both normal progenitors and leukemia cells; and production of immunosuppressive soluble factors and metabolites (64, 65). Antigen presentation by MHC class II genes is reportedly downregulated during relapse following allogeneic stem cell transplantation (ASCT) (66). Although checkpoint blockade is correlated with high mutation and neoantigen burden, the role of checkpoint blockade in AML remains unclear (67). Indeed, AML is considered to present a low mutational burden and thereby low immunogenicity. Nevertheless TP53-mutated AML should be associated with an increased mutation burden and efficacy of ASCT, indicating sufficient immunogenicity (65). In addition, bone marrow T-cells overexpress PD1 and CTLA-4 while LAG-3 and T-cell Immunoglobulin Mucin-3 (TIM-3) are not overexpressed (57, 65, 68). Type I and II interferons also induce the expression of PD-L1/2 in AML blasts (69, 70). TIM-3, CD84, and LAG-3 are increased at the RNA and protein levels in leukemic marrow (58). Indeed TIM-3 is involved in an autocrine stimulatory loop in AML LSCs. Besides, MDSC and M2-polarized macrophages are associated with a poor prognosis, in a manner similar to that of high proportions of M1-polarized macrophages (57, 65). Fatty acid and lipid mediators also modulate the leukemia microenvironment *via* immune signaling (71). Indeed, PGE2 a lipid mediator, has been shown to promote tumor progression *via* the induction and maintenance of MDSC *via* PD-L1 inducing expression on tumor associated macrophages and MDSCs (72).

By contrast, in AML, cytotoxic T-cells (Teff) fail to eliminate leukemic blasts and become senescent *via* the activity of immunosuppressive cells, such as Treg (73). Treg levels are correlated with the response to chemotherapy wherein the lowest levels have been observed during hematopoietic recovery following chemotherapy (74). In addition, an integrated, quantitative immune cell and phenotype profile of AML patients reflects a poor prognosis with a high proportion of M1-polarized macrophages and FOXP3^+^ helper T-cells, with a similar outcome between bone marrow and peripheral blood samples (57). Two major immunologic divergent clusters were identified and correlated with age, T-cell receptor (TCR) clonality and survival. Elderly cluster represents higher TCR clonality, higher OX40 expression in cytotoxic and Treg and higher CD45RO^+^ T-cells, as well as lower naïve and central memory CD27^+^, memory CD25^+^ and late-stage CD57^+^ cytolytic T-cells. Clonality trends were found to be associated with complex karyotypes and higher ELN risk but failed to show a significance due to the insufficient number of patients studied, while an association between T-cell lineage and AML gene mutations has been reported (15, 57). Notably, although FLT3-mutated AML reportedly displays dendritic cells and Treg expansion, Teff are able to perform effector functions in the absence of an enriched Treg population (75). In addition, AML blasts may also present HLA-E and suppress NK cell functions *via* NKG2A activation (76). NK cells express NKG2D, an activator receptor for NKG2D ligands (NKG2DL), such as MHC class I polypeptide-related sequence A and B (MICA/B) and UL16-binding protein (ULBP), that are expressed on AML blast but could be shedding (77).

## Current Therapies and Perspectives

### Standard Cytotoxic Chemotherapies Remain the Main Treatment for the Induction Phase

For decades, the standard therapy of intensive induction chemotherapy 7 + 3 regimen has been the backbone for younger and fit patients. This therapy involves a combination of continuous infusion of cytarabine (100 or 200 mg/m² per day) for 7 d and anthracycline treatment (daunorubicin 60 mg/m² per day) for 3 d (1, 2, 4, 78). Despite a complete remission rate of 60%–80% in younger patients and 40%–60% in older patients, the rate of overall survival (OS) is lower, approximating 40% (78). This highlights the critical issue pertaining to a relapse of leukemia following an initial response. For younger patients who are not undergoing ASCT, a consolidation therapy has been proposed since 1994 (78). Intermediate cytarabine dose (IDAC) consolidation is recommended for younger and older patients who are not undergoing ASCT (2). For high-risk older patients, the effect of consolidation is unclear. Interestingly anthracycline-based chemotherapy induces damage associated molecular patterns (DAMPs) such calreticulin, HSP70 and HSP90 and anti-tumor immunity with phagocytosis by antigen-presenting cells, TH1 polarization, cytotoxic CD8^+^ T-cells and NK cells activation leading to immunogenic cell death (79). Thus chemotherapy, particularly the 7 + 3 regimen, triggers immunologic response against leukemic cells. The prospects of adding a third drug such as the purine analog, fludarabine or clofarabine, have been investigated (78). Similar to clofarabine, fludarabine together with the 7 + 3 regimen appears to induce a better response rate in intermediate-risk AML, but OS in younger patients is not improved while toxicity is increased (80–82).

### Targeting Epigenetic Alterations

Elderly or “unfit” patients, with multiple comorbidities who are not eligible to receive standard intensive chemotherapy have few options available. In such cases, hypomethylating agents (HMA), such as azacytidine and decitabine, are mainly used. Low dose cytarabine (LDAC) may also be used (2). Better responses to HMA have been reported with certain subsets of AML, such as azacytidine and TET2 mutation (83) or decitabine and TP53 mutation (84). DNA demethylation in myeloid neoplasms reactivates genes and leads to differentiation. Azacytidine can inhibit RNA methyltransferase DNMT2 and induce RNA demethylation (68, 85). In addition, HMA may recalibrate the immune microenvironment, *via* promoting anti-tumor response by upregulation of the antigen presentation pathway, TH1 and TH17 polarization, cytotoxic CD8 T-cells activation, Treg function reduction and immune checkpoint blockade (64, 86). Conventional HMA exhibits a complete response (CR) rate above 30% with an overall survival (OS) similar to that of a conventional care regimen (87). A novel HMA, Guadecitabine (SGI-110), has been highlighted in a phase 1/2 study by way of a composite CR rate associating CR and CRi (CR/CRi, CR and CR with incomplete platelet recovery and incomplete neutrophil recovery), between 50%–59% (88). Further investigations are ongoing (NCT02348489). Acute promyelocytic leukemia (APL) is considered separately from non-APL. AML, prior to implementation of specific pathophysiology and risk stratification and response, is associated with high cure rates when all-trans retinoic acid- (ATRA) and arsenic trioxide- (ATO) containing therapy is utilized (89). Nevertheless ATRA and ATO have demonstrated immune modulatory activity which exerts an effect on CD4^+^ and CD8^+^ T-cells and dendritic cells development, and also enhances NK cell mediated cytolytic activity *via* NK ligand upregulation (65, 90). In this regard, ATRA and ATO are being investigated together, or in combination with novel therapies, in IDH1/2-mutated AML (65, 91). Of note, glasdegib, an inhibitor of the smoothened multi-transmembrane (SMO), a component of the Hedgehog pathway, combined with LDAC, which was approved for newly-diagnosed AML in the elderly, who are ineligible to receive standard induction chemotherapy, has shown an increase in the OS rate (8.8 months versus 4.9 months in LDAC only) (92, 93).

### Allogenic Hematopoietic Stem Cell Transplantation as Curative AML Treatment and First Immunotherapy

For “fit” patients with ELN-2017 intermediate or high risk disease (2), performing allogenic hematopoietic stem cell transplantation (ASCT) during first complete response (CR1) offers durable remission and long term survival, whereas no ASCT has a hazard ratio (HR) of 0.80, when weighed against treatment related morbidity (TRM) and mortality (94). Alternative ASCT, such as haploidentical ASCT, in CR1 confers lower leukemia-free survival (LFS) with a HR of 1.74 for intermediate-risk, whereas no differences were seen in high-risk disease, compared with HLA-matched unrelated donor (MUD) (95). Other alternative ASCTs, such as cord blood unit (UCB) or mismatched unrelated donor (MMUD), show LFS comparable to haploidentical ASCT (96). MMUD is still the best alternative option for patients without related HLA-matched donors during CR1 to intermediate-risk AML (97). However, although haploidentical ASCT is an alternative ASCT with debatable LFS outcomes (98, 99), recent results pertaining to myeloablative conditioning (MAC) were at least, either equal or better than MMUD ASCT (100, 101), that led to a prospective investigation of MMUD versus haploidentical ASCT (NCT03655145). Notably, in CR1, MAC regimen is associated with a greater risk for infection, particularly bacterial infection, before day 100, compared with reduced-intensity of non-myeloablative conditioning (RIC/NMA) (102). In addition, MAC and RIC/NMA of various regimens induced different LFS, OS and non-related morbidity and mortality rates. Those outcomes are associated with graft-versus-host disease (GVHD), toxicity and infectious complications (103–106). Then the European Bone Marrow Transplantation society (EBMT) has published a transplant conditioning intensity score (TCI) that provides a better RIC/MAC classification (107). As same, the EBMT has published recommendations about prophylaxis and management of GVHD (108). Although, MAC regimens promote better outcomes in high-risk AML, these are associated with greater toxicity and infection risk, whereas RIC/NMA regimens induce higher GVHD rates (105, 106, 109). Currently MAC regimens are recommended for young patients while RIC regimen are recommended for older patients with comorbidities (2). GVHD prophylaxis is critical for the successful application of ASCT, but still needs to be standardized (110). Prophylactic donor lymphocyte infusion (DLI) is offered following ASCT. Retrospectively it seems to improve OS only in high-risk AML and/or ASCT beyond CR1 (111). Relapse following ASCT benefits from DLI or a second ASCT *via* a MAC or RIC regimen without differences in the 5-year OS. However, relapses less than 6 months after primary ASCT inducing a worse OS, regardless of the treatment prescribed have been observed (112, 113). The use of azacytidine for maintenance following ASCT has been proposed. Although well tolerated, azacytidine has failed to demonstrate improvement of OS in phase 3 studies (114). Moreover, immune pressure may lead to clonal evolution as recently described after ASCT (115). A deviation of the trajectory of leukemia clonal evolution was suggested that allows to escape immune control by different mechanisms, such as genomic HLA loss. In addition, several deregulations of costimulatory ligands on AML blasts (PD-L1, B7-H3, or CD80) have been also highlighted with concomitant changes in donor T-cells after ASCT. These immune control escape mechanisms could be interesting to target in intermediate- and high-risk AML. Use of IFNγ and immune checkpoint blockade therapies might overcome this two evade mechanisms, respectively.

## New Available Therapiesand Perspectives

### Improvement of Cytotoxicity and Specificity of Chemotherapy Toward AML Cells

Recently, several drugs have been approved by the FDA for AML therapy as follows: a liposomal formulation of daunorubicin and cytarabine (CPX-351); the anti-CD33 antibody drug conjugate gemtuzumab ozogamicin (GO); the IDH1/2 mutant inhibitors ivosidenib and enasidenib; the FLT3 inhibitor midostaurin in combination with chemotherapy and gilteritinib; and the BCL2 inhibitor venetoclax in combination with HMA (116). Daunorubicin-cytarabine liposome, with a molar ratio at 5:1, reportedly improves the response rate compared to the standard 7 + 3 regimen, with an overall response rate (ORR) of 47.7% versus 33.3%, p=0.016, in 60/65 year-old patients who are newly-diagnosed with MRC-AML and tAML (117). However, OS is not improved while hematological toxicities are enhanced. Nevertheless, pharmacologically this formulation allows longer half-life and greater AUC, thereby prolonging exposure to leukemic cells that may reduce multidrug resistance (118), and overcome Pgp-mediated drug resistance (116).

The anti-CD33 antibody drug conjugate gemtuzumab ozogamicin (GO) was finally approved in 2017 by the FDA following a decade of controversy (119). CD33 (or Siglec 3) is a transmembrane receptor expressed in myeloid cells but not in normal HSCs that are widespread among AML blasts (>90%) and many AML precursors (120). When GO binds CD33, this receptor internalizes GO which then releases ozogamicin, a calicheamicin derivative, under the acidic conditions of lysosomes. GO was initially developed as a form of monotherapy involving a single 9 mg/m² dose during the first recurrence of CD33^+^ AML. CR recovery is 13% with a median recurrence-free survival of 6.4 months. Main complications are grade 3 and 4 neutropenia (98%), thrombopenia (99%), and a few venoocclusive diseases (VOD, 0.9%) (121). Several studies that were performed did not report an improvement in OS or significant toxicities (78, 119). A better understanding may be obtained from the Acute Leukemia French Association (ALFA) 701 study which used a GO fractionated 3 mg/m² dose on days 1, 4, and 7 together with a standard 7 + 3 regimen (122). A meta-analysis of GO together with induction chemotherapy concluded that addition of GO significantly reduced the risk of a relapse (HR 0.81, p=0.0001) and improved the 5-year OS (HR 0.90, p=0.01) (123). At 6 years, the survival benefit is especially apparent with favorable and intermediate risks. In addition, in NPM1 mutated AML, GO and chemotherapy regimen was associated with fewer relapses following CR (124). CD33 expression itself does not appear to exert a marked effect on AML treatment outcome. However, Core Binding Factor (CBF) AML, which appears to benefit from GO treatment, exhibits blasts with relatively low CD33 levels. This could be explained by high chemosensitivity or CBF AML which arises from CD33^+^ precursors (120). Notably, CD33 binding could be impaired by the germline CD33 single-nucleotide polymorphism, rs12459419, which is associated with the expression of an alternatively spliced variant of CD33 (125). The risk of developing VOD is a major toxicity concern. The risk is lower with GO doses no greater than 3 mg/m², but increases in heavily treated patients (119). However, GO prior to ASCT does not seem to increase VOD risk after ASCT (126). A similar VOD risk is reported with inotuzumab ozogamicin, an anti-CD22 antibody with the same conjugate drug, suggesting that CD33 independent toxicity may occur (127). Nevertheless, a new anti-CD33 antibody conjugated with pyrrolobenzodiazepine dimer instead of calicheamicin (SGN-CD33A) indicates that targeting CD33 may also contribute to VOD (128). Occurrences of VOD stop its development. A different method for targeting CD33 involves using chimeric antigen receptor T-cells (CAR T-cells) that exhibit potent preclinical activity against human AML (129). However, the efficacy of this method against refractory/relapse (R/R) AML is still under investigation (130). Safety assessment, particularly concerning the VOD risk, will be vital.

### Targeted Therapies

#### IDH1/2-Targeting in Relapse/Refractory AML or Newly Diagnosed Older Non Eligible AML

Blockade of IDH1/2-mutated enzyme by inhibitors induces differentiation of malignant blasts (16). IDH1/2 mutations induce the production of oncometabolite R-2-Hydroxyglutarate (R-2-HG), which is responsible for a blockade in terminal differentiation *via* calcium influx reduction, NFAT translocation and proliferation suppression (131, 132). Enasidenib and ivosidenib, inhibitors of mutant IDH2 and IDH1 respectively, have recently been approved by the FDA for IDH2-mutated and IDH1-mutated AML. Treatment consisting of the single agent, Enasidenib, resulted in a CR of 19.6% and an overall response rate (ORR) of 38.8% with a median OS of 8.8 months in R/R IDH2-mutated AML (17, 133). CR is achieved *via* the clearance of mutant-IDH2 clones, but interestingly functional mutant-IDH2 neutrophils are detected, suggesting a conversion from undifferentiated to differentiated myeloid cells (18). In addition, enasidenib specific toxicity profile is corroborated by IDH inhibitor-associated differentiation syndrome (IDH-DS) (17). Non-responding patients are significantly associated only with FLT3 baseline mutations. Association with NRAS and other MAPK pathway effector mutations remain unclear, but may be associated with non-response to enasidenib (18, 133). Interestingly, R-2-HG diminution may also reduce the paracrine hyperleukocytosis and leukemogenesis effect on IDH2 wild-type clones (134). On the other hand, ivosidenib, a single agent used against R/R IDH1-mutated AML (19), which has been newly-recognized as ineligible for standard chemotherapy against IDH1-mutated AML (135), showed a CR/CRi rate of 30.4% and 42.4%, respectively, and an ORR of 41.6% and 54.5%, respectively, with a median OS of 8.8 months and 12.6 months, respectively. The toxicity profile is similar, and IDH-DS has been described. Furtherer associations with azacytidine or venetoclax are under investigation (136, 137).

#### Active Developments in the Area of FLT3 Inhibitors

Several FLT3 tyrosine kinase inhibitors have been developed in the last few years with variable pharmacological and clinical profiles. FLT3 inhibitors are divided into first generation multi-kinase inhibitor (including sorafenib, lestaurtinib, midoastaurine) and next generation inhibitors (including quizartininb, crenolitinib, gilteritinib) (138). Many trials are ongoing investigating FLT3 inhibitors alone or in combination with standard chemotherapy or HMA, in first line or in relapse. The broad-spectrum FLT3 inhibitor midostaurin has been recently approved in combination with standard of care chemotherapy. Midostaurin, used as a single agent, has reduced or eliminated FTL3-mutated and FTL3-wild-type blasts, but showed limited effect on BM blasts as well as a short therapeutic duration (139). This suggests the necessity for combining midostaurin with chemotherapy. Midostaurin in association with daunorubicin and cytarabine induction and consolidation chemotherapy, or alone in maintenance, has demonstrated a median OS of 74.7 months as opposed to 25.6 months in the placebo arm, with a HR for death of 0.78 (p=0.009), and a 4-year OS rate of 51.4% and 44.3%, respectively (140). ASCT, which was performed at the discretion of the investigators in 57% of patients, has shown a 4-year OS rate of 63.7% in the midostaurin arm as opposed to 55.7% in the placebo arm but without significance (p=0.08).

On the other hand, sorafenib used in association with azacytidine in ineligible *de novo* FTL3-mutated AML and in R/R FLT3-ITD-mutated AML, has shown a CR rate of 26% and 27% respectively and a CR/CRi rate of 70% and 43% (141, 142). Association with standard induction chemotherapy improved the median EFS to 21 months versus 9 months in the placebo arm, but was accompanied by a significant increase in toxicity (143). In maintenance therapy following ASCT, sorafenib permitted a greater relapse-free survival (RFS) than the placebo arm, with a 2-year RFS rate of 85% versus 53.3%, respectively (144). Despite interesting results, except for significant toxicity and absence of a proven OS benefit, sorafenib is not used as a frontline drug that is combined with standard intensive chemotherapy (145). However, regardless of the absence of appropriate clinical trials, prospective large-scale studies have made sorafenib a preferred option for maintenance following ASCT. Despite the development of FLT3 inhibitors, the EMBT Acute Leukemia Working Party recommends performing ASCT in their 2017-ELN risk stratification, but with the accompaniment of post-transplantation FLT3 inhibitor maintenance for at least 2 years (146). Of note in both FLT3-mutated and FLT3-wild type AML, midostaurin has demonstrated immune-modulatory effects as shown by a reduction in the CD4^+^ CD25^+^ cells proportion, mRNA levels of FOXP3 and IL-10 and TNFα levels (147). This decrease in Treg might be possibly linked to FLT3-ITD inhibition.

Gilteritinib has been recently approved for single use in R/R FLT3-mutated AML. Compared with chemotherapy, gilteritinib has demonstrated a greater median OS (9.3 months versus 5.6 months with HR of death of 0.64), CR, and CR/CRI (21.1% versus 10.5% and 34% versus 15.3% respectively), whereas its incidence of exposure-adjusted events was lower (19.24 versus 42.44 respectively); (148). Interestingly, gilteritinib shows continuous target inhibition despite FLT3-D835 mutation, whereas sorafenib do not show such inhibition. The use of other FLT3 inhibitors still needs to be determined. Despite the highest FLT3 inhibition and an improvement of OS rate (6.2 months versus 4.7 month for salvage chemotherapy), quizartinib is associated with marginal clinical benefits and significant toxicity levels (149). For this reason quizartinib did not receive FDA drug approval yet (145). A novel FLT3 inhibitor, crenolanib, presents a FLT3 inhibition despite FLT3-D835 mutation, that might be interesting in relapse, but is currently still under investigation (150).

#### Apoptosis Regulators as Novel Anti-Leukemic Strategies

Venetoclax, an inhibitor of BCL2 apoptosis regulation, which was initially used as a single agent in AML relapse, has shown an ORR of 19% in a preclinical study. In elderly, untreated patients with AML who are ineligible for standard induction chemotherapy, venetoclax combined with HMA resulted in a CR/CRi rate of 67%, and a CR/CRi of 91.5%, in NPM1-mutated AML (151). In TP53-mutated AML, this combination was promising, with CR/CRi of 47% and a median OS of 7.2 months. However, 70% of patients have discontinued the treatment due to disease progression. Recently a phase 3 study confirmed CR/CRi rate improvement with venetoclax adjunction to HMA (66.4% versus 28.3%, p<0.001) with an increasement of median OS (14.7 months versus 9.6 months, HR for death 0.66%, p<0.001) (152). Similarly, in elderly patients with newly-diagnosed AML who are ineligible for standard induction chemotherapy, a combination of venetoclax and LDAC was associated with a CR/CRi of 54% (153), while, in a phase 3 study, the same combination showed a median OS of 8.4 months as opposed to 4.1 months with LDAC alone (HR 0.70, p=0.04); (154). NPM1- or IDH1/2-mutated AML have been associated with higher CR/CRi rate (89% and 72%, respectively) in contrast to TP53- or FLT3-mutated AML (30% and 44% respectively). Venetoclax resistance may be driven by MCL-1 overexpression (155). Further suggested combinations, including drugs targeting XPO1, CDK9, MDM2, or MEK, await determination *via* other studies (156). The association between Venetoclax and chemotherapy is also under investigation (157). Interestingly, response in NPM1-mutated AML may be linked to tumor-specific immunity recovery of T-cells (65) and HLA-presentation in the mutant protein of NPM1 (NPM1c) (158). NPM1c-targeting has also been proposed (159). In addition, nuclear relocalization of NPM1c, induced *via* XPO1 inhibition by selinexor, promotes arrest of growth and differentiation (160). Moreover, a retrospective study of elderly NPM1-mutated AML patients with positive NPM1-measurable residual disease (MRD), indicated that initiation of venetoclax, following induction and consolidation of chemotherapy together with azacytidine or LDAC, induces rapid clearance of residual NPM1-blasts (161). Of note, the tumor suppressor p53, deficient in TP53-mutated AML, is associated with pro-apoptotic activity following DNA damage, and inhibits BCL2 directly as well as indirectly (155). Although, BCL-2 inhibitors in TP53-mutated AML appear to be promising, further investigations are required.

## Therapies in Development

### Epigenetics and Tyrosine Kinases Inhibitors

**Figure 1:**
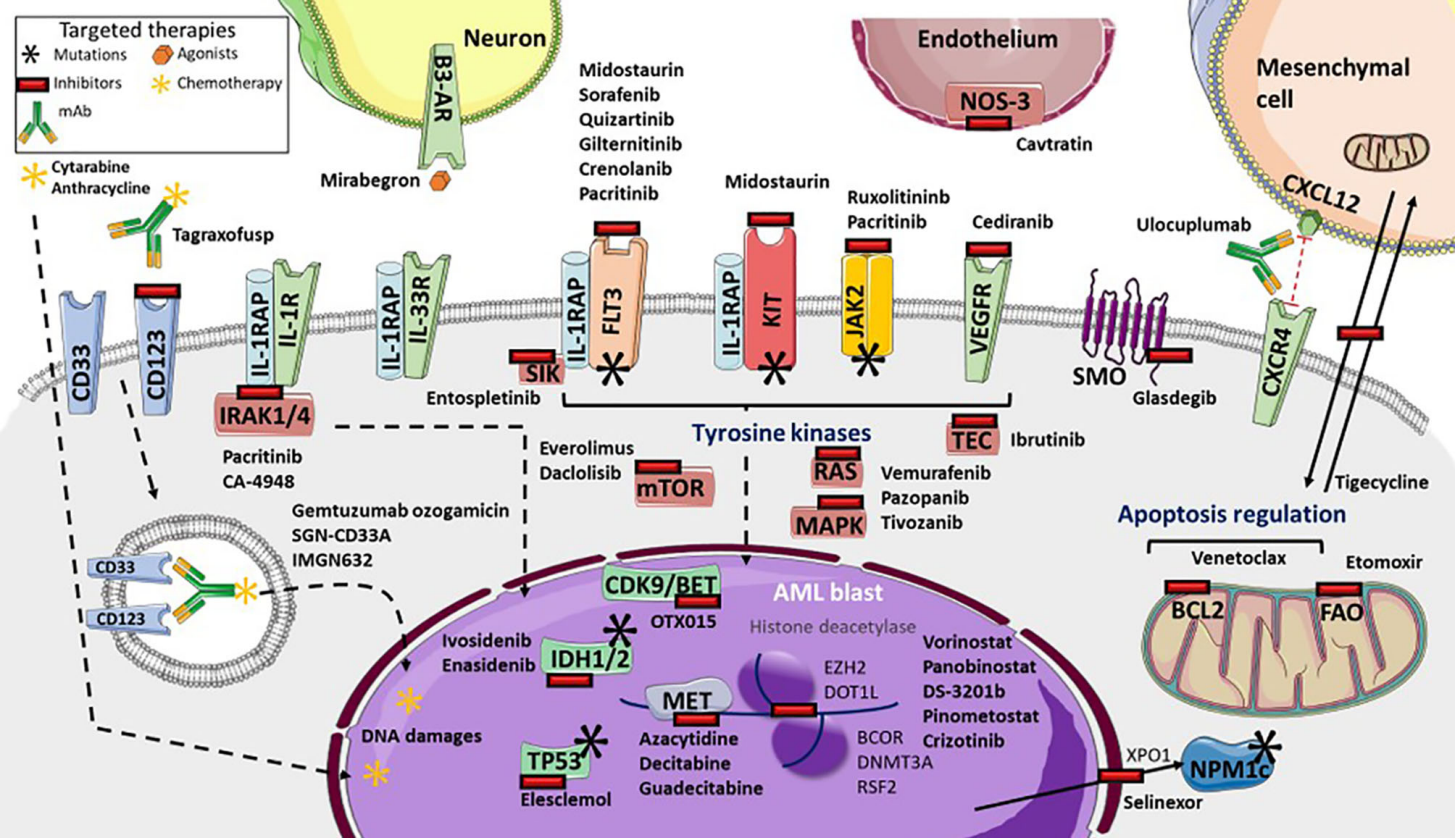
Targeted therapies available and in development in Acute Myeloid Leukemia (AML). Many tyrosine kinases inhibitors are either currently available or in development. These include: Fms-Like Tyrosine Kinase 3 (FLT3) inhibitors (such as sorafenib, quizartinib, gilteritinib, crenolanib), pan-kinase inhibitors (including midostaurin), FLT3 and KIT inhibitors (including pacritinib), Janus Kinase-2 (JAK2) and interleukine-1 Receptor (IL-1R) Associated Kinases 1/4 (IRAK1/4) inhibitors, the IRAK4 inhibitor Ca-4948, the JAK2 inhibitor ruxolitinib, NRAS, KRAS and MAP Kinases (MAPK) inhibitors (such as vemurafenib, pazopanib, tivozanib), mTOR inhibitors (everolimus and dactolisib), TEC kinases inhibitors (including ibrutinib), and vascular and endothelial growth factor receptor (VEGFR) inhibitor cediranib. Several targeted-drugs are available, or in development, for transcription factors: ivosidenib, an Isocitrate Deshydrogenase-1 (IDH1) inhibitor, enasidenib, an IDH2 inhibitor, azacytidine, decitabine, guadecitabine as hypomethylated agents, the histone deacetylase vorinostat and panobinostat, DS-3201b, a zeste 2 polycomb repressive complex 2 subunit (EZH2) inhibitor, pinometostat, a DOT1-like histone lysine methyltransferase (DOT1L) inhibitor, crizotinib, the Bcl6 Corepressor (BCOR) inhibitor, OTX015, a cyclin-dependent kinase 9/bromodomain and extraterminal (CDK9/BET) inhibitor, and elesclomol, a TP53 inhibitor. Selinexor inhibits the XPO1 exporter, which inhibits leukemic activity of mutated NPM1 proteins. Glasdegib inhibits smoothened multi-transmembrane (SMO), a member of the Hedgehog pathway. Tagraxofusp inhibits CD123, whereas IMGN632 transports chemotherapy through CD123 internalization, as does gemtuzumab ozogamicin and SGN-CD33A through CD33. Venetoclax inhibits the BCL2 anti-apoptotic protein. Several microenvironment targeted drug are in development: etomoxir inhibits fatty acid oxidation metabolism, tigecycline inhibits mitochondrial heterocellular transfer, thus inhibiting drug resistance exchange, ulocuplumab inhibits CXCR4/CXCL12 interaction from inducing leukemia myeloid cell migration, mirabegron, an agonist for sympathetic neuropathy β3-adrenergic receptor (β3-AR), and cavtratin, the NO synthase inhibitor.

Gene expression profiling, which helps identify new targets as well as drug resistance, provides information that is valuable for the development of therapeutic strategies (**Figure 1**) (9, 82). Recent strategies advocate inhibition of other epigenetic modifiers, such as the enhancer of zeste homolog 2 (EZH2) (162), lysine demethylase 1A (KDM1A or LDS1) (163) and DOT1-like histone lysine methyltransferase (DOT1L) (164). The MLL subset interacts with DOT1L and the bromodomain and extra-terminal (BET) family member, bromodomain-containing 4 (BRD4), leading to potent inhibition (165, 166). The OTX015 BET inhibitor is currently being clinically investigated following a phase 1 study (167). By contrast, vorinostat and panobinostat histone deacetylase inhibitors, that were initially promising, either alone or with HMA or cytarabine regimen, have failed to demonstrate benefits in AML (168–170). Several trials of multiples of other epigenetic targets, such as DS-3201b or pinometostat, are ongoing (171). In addition, many of the several genes that were found to be correlated with drug sensitivity or resistance, are from the BEAT cohort study (9). TP53 mutations or NRAS and KRAS mutations cause broad patterns of drug resistance, but TP53 mutations trend more sensitivity to elesclemol, in a manner similar to that of NRAS mutation with MAPK inhibitors, such as vemurafenib, pazopanib, or tivozanib. However, sensitivity of KRAS mutations is lower. Mutations in RUNX1 are correlated with sensitivity to PIK3C and mTOR inhibitors, such as dactolisib, and to cediranib, a multi-kinase VEGFR inhibitor. Everolimus is also being investigated (172). Similarly, FLT3-ITD mutations with or without NPM1 mutation, are significantly sensitive to ibrutinib, an inhibitor of BTK and TEC family kinases, as well as entospletinib, an inhibitor of spleen-associated tyrosine kinase (SYK). SYK interacts with FLT3 (173), and thus targeting SYK with entospletinib may be effective in FLT3-mutated AML and is currently under investigation (NCT02343939, NCT03135028, NCT03013998) (**Table 2**). Lastly, BOCR and RUNX1 mutations are sensitive to JAK kinase inhibitors, such as ruxolitinib, whereas BCOR mutations, either alone or in association with DNMT3A or RSF2 are not. However BCOR mutations alone show sensitivity to the multi-kinase inhibitor crizotinib (9). Interestingly, targeting the IL-1 pathway with the multi-kinase inhibitor, pacritinib, which inhibits IRAK1/4, FLT3, and JAK2, highlights robust sensitivity despite adaptive resistance to therapies and significant AML cell death (174, 175). Further investigations are ongoing (176). IRAK1 inhibitor association with venetoclax also appears to be interesting (47). In addition, the IRAK4 inhibitor, CA-4948, is under investigation for its effect on AML (NCT04278768).

**Table 2:** Targeting drugs under investigations (based on www.clinicaltrials.gov at 05/25/2020).

Targeting drug	Name	Spectrum	Results	Status	Clinical Trials
*Targeted cytotoxic drug*					
CD33	gemtuzumab ozogamicin	CD33+	Relapse risk reduction (HR 0.81), 5 year-OS improvement (HR 0.90)	FDA approval	25 recruiting studies in association
	SGN-CD33A (vadastuximab talirine)	CD33+	Higher rate of death	*Terminated*	*NCT02785900*
	lintuzumab-Ac225	CD33+	Under investigations	Phase 1 & phase 1/2	NCT03441048, NCT03867682
CD123	IMGN632		Under investigations	Phase 1/2	NCT04086264, NCT03386513
*Epigenic-directed therapies*					
IDH1	ivosidenib	IDH1^mut^	R/R AML: CR/CRi 30.4%, ORR 41.6% (phase 1)	FDA approval	9 recruiting studies alone or in association
IDH2	enasidenib	IDH2^mut^	R/R AML: CR 19.6%, ORR 38.8% (phase 1/2)	FDA approval	12 recruiting studies alone or in association
HDAC	panobinostat		Limited benefit	Phase 3	NCT04326764
	vorinostat		Limited benefit	Phase 1 & phase 1/2	NCT03263936, NCT03843528, NCT03878524, NCT03842696
	belinostat		Under investigations	Phase 1	NCT03772925
	entinostat		Under investigations	Phase 2	NCT01305499
EZH2	DS-3201b		Under investigations	Phase 1	NCT03110354
DOT1L	pinometostat	MLL^mut^	Under investigations	Phase 1/2	NCT03724084, NCT03701295
KDM1A	tranylcypromine		Under investigations	Phase 1	NCT02273102
	INCB059872		Under investigations	Phase 1	NCT02712905
BET	OTX015	MLL^mut^	*Study withdrawn*	*Phase 1*	*NCT01713582, No further study*
	GSK525762		Under investigations	Phase 2	NCT01943851
BCOR	crizotinib	BCOR^mut^	Under investigations	Phase 2	NCT02638428
BCL2	venetoclax	NPM1^mut^ IDH1/2^mut^TP53^mut^	In association to HMA in AML ineligible: CR/CRi 67%	Phase 3	56 recruiting studies in association
TP53	elesclomol	TP53^mut^	Under investigations	Phase 1	NCT01280786
XPO-1	selinexor	NPM1^mut^	Under investigations	Phase 1	NCT02091245, *NCT02093403, NCT02299518*
*Kinase inhibitors*					
FLT3	midostaurin	FLT3^mut^	Front line FLT3^mut^-AML: OS 74.7 months (HR 0.78)	FDA approval	13 recruiting studies alone or in association
	sorafenib	FLT3^mut^	Largest prospective studies but significative toxicities	Phase 2	12 recruiting studies in association
	gilteritinib	FLT3^mut^	Phase 3: OS 9.3 months vs 5.6 months (HR=0.64), CR/Cri 34%	FDA approval	9 recruiting studies in association
	quizartinib	FLT3^mut^	Limited benefit	FDA refusal	9 recruiting studies in association
	crenolanib	FLT3^mut^	Sensibility despite FLT3-D835 mutation	Phase 2 & 3	NCT03258931, NCT03250338, NCT02400255
MAPK	vemurafenib	NRAS^mut^	Under investigations	Phase 1 & 2	NCT03878524, NCT02638428
	pazopanib	NRAS^mut^	Under investigations	Phase 2	NCT02638428
	tivozanib	NRAS^mut^	Sensibility	Pre-clinical	None
mTOR	everolimus	RUNX1^mut^	Under investigations	Phase 1/2	NCT02109744
PIK3C-mTOR	dactolisib	RUNX1^mut^	Sensibility	Pre-clinical	None
VEGFR	cediranib	MLL^mut^	*No confirmed responses*	*Phase 2*	*NCT00475150, no further study*
BTK/TEC	ibritinib	FLT3^mut^ ± NPM1^mut^	Under investigations	Phase 2	NCT02351037, NCT03267186
SYK	entospletinib	FLT3^mut^	Under investigations	Phase 1/2	NCT02343939, NCT03135028, NCT03013998
JAK2	ruxolitinib	JAK2^mut^	Under investigations	Phase 1/2	NCT03558607, NCT02257138
	ruxolitinib + decitabine	BCOR^mut^	Under investigations	Phase 2	NCT04282187
	ruxolitinib + venetoclax	RUNX1^mut^	Under investigations	Phase 1	NCT03874052
IRAK1/4-FLT3-JAK2	pacritinib		*Anti-AML activity* *but stopped due to financial constraints*	*Terminated*	*NCT02532010*
IRAK4	CA-4948		Under investigations	Phase 1	NCT04278768, NCT03328078

Overview of targeting drug studied in AML. Mut, mutation. R/R AML, relapse and refractory AML. OS, overall survival. CR, complete response. CRi, complete response with incomplete hematological recovery response. ORR, overall response rate. FDA, Food and Drug Administration.

### Immunotherapies

#### Monoclonal and Bispecific T-Cells Engagers (BiTE) Antibodies as Form of Passive Immunotherapy

**Figure 2:**
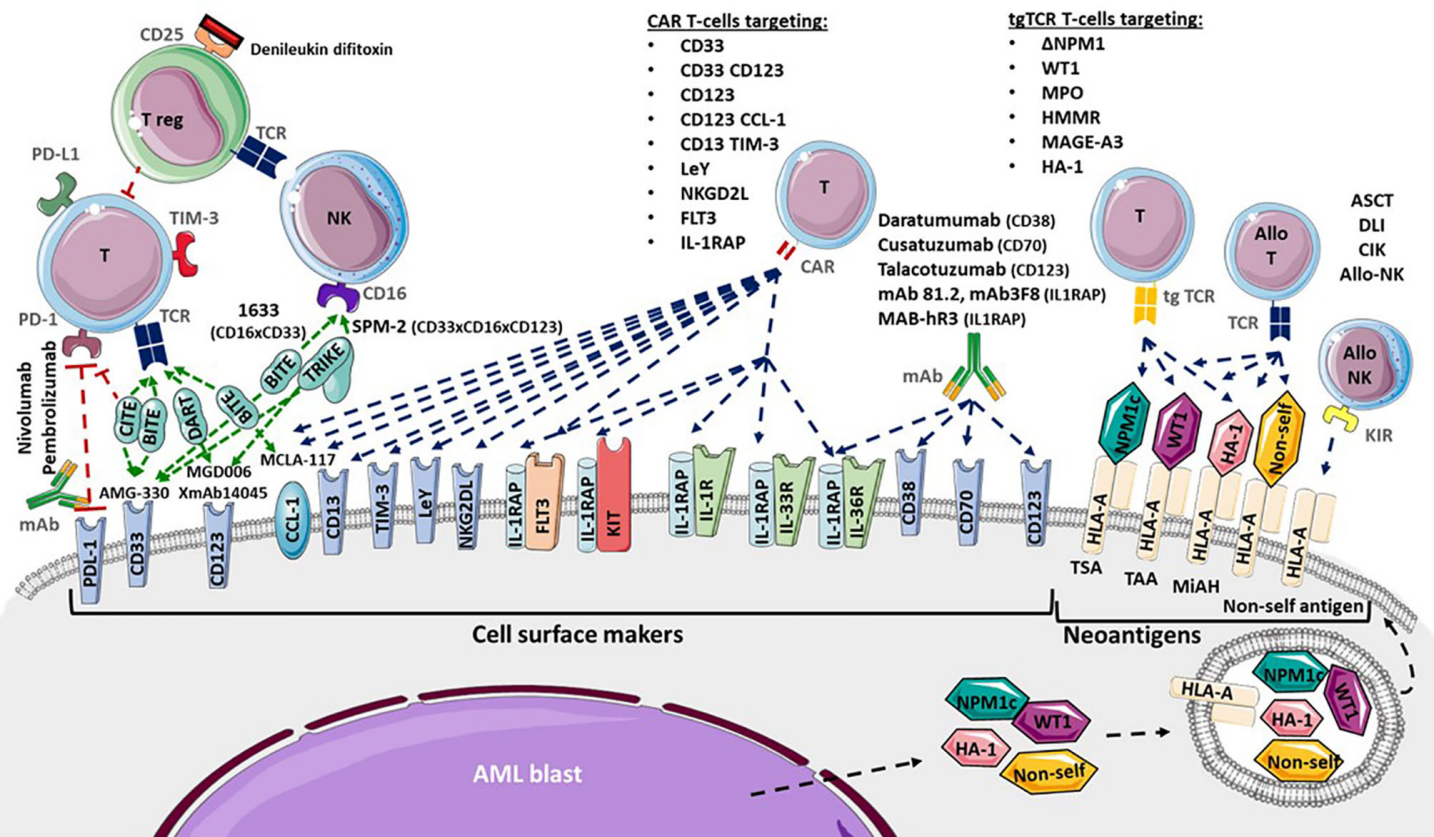
Adoptive immunotherapies available and in development for Acute Myeloid Leukemia (AML). Cytotoxicity functions of T-cells (T) and NK-cells (NK) are investigated in several ways. Allogenic hematopoietic Stem Cell Transplantation (ASCT), Donor Lymphocytes Infusion (DLI), Cytokine-induced Killer cells and donor NK-cells infusion provide allogenic T-cells (allo T) and NK-cells (allo NK) targeting neoantigens including Human Leukocyte Antigen (HLA) Major Histocompatibility Complex (CMH) mismatch, Killer-cell Immunoglobulin-like Receptor (KIR) mismatch, Minor Histocompatibility Antigen (MiHA), Tumor Specific Antigen (TSA) and Tumor Associated Antigen (TAA). Transgenic T-cell receptor (tgTCR) T-cells could target the nucleophosmin-1- (NPM1) mutated antigen (ΔNPM1), Wilms’ Tumor 1 (WT1), Myeloperoxidase (MPO), Hyaluronan-mediated motility receptor- (HMMR/Rhamm), Melanoma Associated Antigen-A3 (MAGE A-3), leukemia-associated minor H antigen 1- (HA-1). Monoclonal antibodies could target CD38 (Daratumumab), CD70 (Cusatuzumab), CD123 (Talacotuzumab) and IL1RAP (mAb 81.2, mAb3F8, MAB-hR3) and induced AML cells lysis. Blockade antibodies could target Program Cell Death 1 (PD-1) and PD-1 ligand (PD-L1). Bispecific T-cells Engagers (BiTE) (AMG-330, XmAb14045, MCLA-117), Dual-Affinity Re-Targeting (DART) (MGD006), Bi- and Tri-specific Killer Engagers (BiKE, TriKE) (1633, SPM-2) and Checkpoint inhibitor T-cell Engager (CiTE) antibodies could engage T-cell targeting toward specific antigens. Debileukin difitoxin could block IL-2 receptor (CD25) and then induce regulatory T-cells (Treg) apoptosis. Chimeric Antigen Receptor (CAR) T-cells could target CD33, CD123, C-type lectin domain family 12 member A (CLEC12A or CCL-1), CD33 and CD123, CD123 and CCL-1 (compound CAR), CD13 and T-cells Immunoglobulin Mucin-3 (TIM-3) (bispecific CAR), CD38, CD44, Lewis Y (LeY), Natural Killer Group 2D Ligand (NKG2DL), B7-H3, Fms-Like Tyrosine Kinase 3 (FLT3), c-KIT (CD117) and interleukine-1 Receptor Accessory Protein (IL1RAP).

Cell surface markers of LSC and AML blasts may be targeted using several strategies, including antagonists, monoclonal antibodies (mAb), CAR T-cells or transgenic TCR (**Figure 2**). Monoclonal antibodies could target one antigen, then induce different response depending on the kind of cells or molecules recruited: direct apoptosis, complement-dependent cytotoxicity, and antibody-dependent cell-mediated cytotoxicity (36). But technological progress has permitted to develop synthetic antibodies capable of several antigen targeting, such as Bispecific T-cells Engagers (BiTE) or dual-bodies. CD33-targeting with GO is currently the most advanced strategy available, but CD33-targeting is still under investigation, particularly to limit VOD, as described previously. Indeed the effect of several mAbs, with or without a conjugated drug, on CD25, CD38, CD44, CD45, CD47, CD70, CD123, CD157, FLT3, IL1RAP, and CCL-1, are being investigated (36, 177). Only a few translations from preclinical models to clinical trials have shown a satisfactory response in AML clinical studies (**Table 3**). In phase 1, anti-CD25, anti-CD44 and anti-CD47 antibodies failed to obtain a significant response (177–179). Only a few minor studies without clear results for anti-CD45 radiolabeled antibodies have been reported. No results have been reported by the clinical trial investigating the anti-CD157 antibody (NCT02353143) as of yet (177). Anti-FLT3 antibody shows potent preclinical activity and is being tested as a single agent for efficacy in R/R FLT3-mutated AML (NCT02864290) (180). The anti-CD70 antibody cusatuzumab has shown a CR/CRi rate of 82% in phase 1 studies (181). Due to positive preclinical results in AML, the anti-CD38 antibody, daratumumab, is currently under investigation for efficacy as a single agent, in phase 2 studies (NCT03067571) (182). Its combination with either azacytidine or ATRA is also under investigation (NCT02807558), but preliminary results appear to be disappointing (183). Interestingly ATRA upregulates CD38 in AML cells (184).

**Table 3:** Antibody based immunotherapies under investigation (based on www.clinicaltrials.gov at 05/25/2020).

Antibody constructions	Name	Results	Status	Clinical Trials
CD123	talacotuzumab	Toxicity	Terminated	NCT02472145
CD38	daratumumab	Under investigations	Phase 1/2	NCT03537599
			Phase 2	NCT03067571
CD44	H90	LSC targeting	Pre-clinical	None
CD47	Hu5F9-G4	Under investigations	Phase 1	NCT02678338, NCT03248479
CD70	cusatuzumab	CR/CRi 82%	Phase 1	NCT04150887,
	SEA-CD70	Under investigations	Phase 1	NCT04227847
CD157	MEN1112	*No report*	*Phase 1*	*NCT02353143*
FLT3	ASP1235	Under investigations	Phase 1	NCT02864290
ILRAP	mAb81.2, mAb3F8	LSC targeting	Pre-clinical	None
	MAB-hR3	LSC targeting	Pre-clinical	None
CD3/CD33 BiTE	AMG-330	Under investigations	Phase 1	NCT02520427
	GEM333	Under investigations	Phase 1	NCT03516760
	JNJ-67371244	Under investigations	Phase 1	NCT03915379
CD3/CD33 Tandem Diabody	AMV564	Under investigations	Phase 1	NCT03144245
CD3/CD33/PD-1 CiTE		AML targeting	Pre-clinical	None
CD3/CD123 DART	MGD006 (flotetuzumab)	Under investigations	Phase 1/2	NCT02152956, NCT04158739
CD3/CD123 BiTE	XmAb14045	CR/CRI 23%	Phase 1	NCT02730312
CD3/CD123 DuoBody	JNJ-63709178	Under investigations	Phase 1	NCT02715011
CD3/CCL-1 BiTE	MCLA-117	Under investigations	Phase 1	NCT03038230
CD16/CD33 BiKE	1633	AML targeting	Pre-clinical	None
CD16/IL-15/CD33 TriKE	161533 (GTB-3550)	Under investigations	Phase 1/2	NCT03214666
CD16/CD33/CD123 TriKE	SPM-2	AML & LSC targeting	Pre-clinical	None

Progress of antibody-based immunotherapy studies in AML. CR, complete response. CRi, complete response with incomplete hematological recovery response. AML, acute myeloid leukemia. LSC, leukemic stem cells. BiTE, Bispecific T-cell Engager. CiTE, Checkpoint inhibitor T-cell Engager. DART, Dual-Affinity Re-Targeting. BiKE, Bispecific Killer Engagers. TriKE, Trispecific Killer Engagers.

Studies pertaining to anti-CD123 unconjugated antibodies, such as talacotuzumab, have been terminated early because of issues related to toxicity (185). However, an interim analysis of anti-CD123 conjugated IMGN632 has shown a CR/CRi rate of 33% (186). A combination of IMGN632 and venetoclax has yielded interesting results in a preclinical model (187). Moreover a CD123 antagonist, the diphtheria toxin IL-3 fusion protein tagraxofusp, indicates potent activity against AML blasts and is under investigation (188, 189). CCL-1- (known as CLEC12A or CD371) targeting combined with CD3-targeting (MCLA-117) using a BiTE antibody is being developed and is at the phase 1 study stage (NCT03038230). In addition, anti-CD3/CD33 BiTE (AMG-330), anti-CD16/CD33 Bispecific Killer Engagers (BiKE) (known as 1633), anti-CD16/IL-15/CD33 Trispecific Killer Engagers (TriKE) (known as 161533), and anti-CD3/CD123 Dual-Affinity Re-Targeting (DART) antibodies (MGD006) are also in development and under investigation for effectiveness in AML (36, 189). The anti- CD3/CD123 BITE XmAb14045 phase 1 showed a CR/CRi rate of 23% (190). Moreover, anti-CD33/CD123/CD16 TriKE is under investigation (191). Another interesting BiTE combination being investigated for overweight PD-1/PD-L1 exhaustion, is PD-1 extracellular domain (PD-EX) blockade in association with anti-CD3/anti-CD33, which is thus a Checkpoint inhibitor T-cell Engager (CiTE) (192).

Lastly, IL1RAP (also named IL-1R3) blockade, using mAb81.2 and mAb3F8, which induces antibody-dependent cellular cytotoxicity and IL-1 signaling blockade in AML and CML cells provides evidence that IL1RAP may be a potent target in AML cells (33, 193). All functions of the IL-1 family (IL-1α, IL-1β, IL-33, IL-36α, IL-36β, and IL-36γ) are attenuated by IL1RAP blockade with the human IL1RAP antibody, MAB-hR3 (194). No increase in IL-6 production was observed, and IL1RAP blockade induced a broader anti-inflammatory activity than that associated with IL-1R1 blockade by IL-1Ra antagonist anakinra. Interestingly, chronic IL-1 exposure drives HSC clonal evolution in AML cells (53), and thus IL1RAP blockade may allow a reduction in bone marrow inflammation associated with AML.

#### T-Cell Transfer Therapy a New Era in Cancer Immunotherapy

##### Chimeric Antigen Receptors (CAR) T-Cells, Leaders in T-Cell Transfer Therapy

A novel adoptive T-cell transfer therapy named Chimeric Antigen Receptor T-cells (CAR T-cells), is recently available in lymphoid neoplasms, using CD19 target with promising results (195–197). T-cells are transduced by a viral supernatant that induces a cell surface chimeric receptor expression. This allows to target a specific antigen expressed at the surface of the tumor cells (198). Afterward, genetically modified T-cells are infused in order to induce a selective tumor lysis after CAR recognition. Thus, several cell surface markers and transgene constructions are investigated for CAR T-cell engineering in AML (130). As compared to mAb, CD33, CD123, CCL-1, FLT3, CD38, CD44 variant 6, and NKG2DL targets are investigated in early phase clinical trial, whereas c-KIT (CD117), B7-H3 (as known as CD276), IL1RAP, and CD13 targets are under preclinical investigation (**Table 4**) (36, 199).

**Table 4:** T-cells immunotherapies under investigations (based on www.clinicaltrials.gov at 05/25/2020).

CAR T-cells	Pre-clinical results	Status	Clinical Trials
CD33	Myeloablative, ASCT requirement	Phase 1	NCT03126864
		Phase 1/2	NCT03971799, NCT01864902
CD123	Myeloablative, ASCT requirement	Phase 1	NCT03796390, NCT03585517, NCT03114670, NCT03766126, NCT04014881, NCT03190278, NCT02159495, NCT04230265, NCT04318678, NCT03672851
		Phase 1/2	NCT04272125, NCT04265963, NCT04109482, NCT03556982
CCL-1	AML and HSC targeting	Phase 1	NCT04219163
CD38	AML targeting	Phase 1/2	NCT04351022
CD44 variant 6	AML targeting	Phase 1/2	NCT04097301
FLT3	Myeloablative, ASCT requirement	Phase 1	NCT03904069
KIT (CD117)	Myeloablative, ASCT requirement	Pre-clinical	NCT03473457
B7-H3	HSC toxicity reduction	Pre-clinical	None
CD13 TIM-3	HSC toxicity reduction	Pre-clinical	None
PD-1	Anti-tumor enhancement	Pre-clinical	None
Lewis Y	Short duration of response, few toxicities	Phase 1	*NCT01716364, no further study*
NKGD2L	Short duration of response, few toxicities	Phase 1	*NCT02203825, no further study*
IL1RAP	LSC targeting	Pre-clinical	NCT04169022
CD33/CD123	AML and HSC targeting	Phase 1	NCT04156256
CCL-1/CD123	AML targeting	Phase 2/3	NCT03631576
CCL-1/CD33	AML targeting	Phase 1	NCT03795779	
CCL-1/CD33 and/or CD123	AML targeting	Phase 1/2	NCT04010877
Muc1/CLL1/CD33/CD38/CD56/CD123	AML targeting	Phase 1/2	NCT03222674
**tgTCR T-cells**	**Pre-clinical results**	**Status**	**Clinical Trials**
NPM1	AML targeting	Pre-clinical	None
WT1	Survival greater than 12 months	Phase 1/2	NCT02895412, NCT01621724, NCT02550535, NCT04284228	
MPO	AML targeting	Pre-clinical	None
HMMR/Rhamm	AML and HSC targeting	Pre-clinical	None
MAGE-A3	AML targeting	Pre-clinical	None
HA-1	AML targeting	Phase 1	NCT03326921

Studies investigating T-cells immunotherapies in AML. AML, acute myeloid leukemia. LSC, leukemic stem cell. HSC, hematopoietic stem cell. ASCT, allogeneic stem cell transplantation.

The two main targets are CD33 and CD123 with several CAR constructions improvement over time. Addition to a 4-1BBz costimulatory transgene in the CD33 CAR construction have shown better antileukemic activity as well as resistance to exhaustion with an increasing central memory comportment (200). Despite use as myeloablative conditioning regimen before ASCT (201, 202), hematopoietic toxicity is a limitation of CD33 and CD123 CAR T-cells, that conducts to investigate different strategies. Thus, two strategies have been developed: modulation of CAR affinity (203), and transient transgene expression (129). Modification by sequence-mutation of anti-CD123 single chain fragment variable (scFv) might conduct to a lesser hematopoietic toxicity (201, 204, 205). Another proposed strategy in order to reduce hematopoietic toxicity is the use of an transiently expressed ARN to induce self-limiting activity against AML cells (129). Inactivation of the *CD33* gene in HSCs prior to transplantation was suggested in order to prevent CD33 induced hematopoietic toxicity of CAR T-cells (206). Yet, CD123 CAR T-cells in combination with ASCT could be an interesting strategy for treating R/R AML patients (189, 202).

Other targets are investigated in the purpose to reduced hematopoietic toxicity. CCL-1 CAR T-cells show interesting preclinical efficacy on LSC and AML blasts without HSC toxicity (207, 208). FLT3 or CD117 CAR T-cells are also cytotoxic against LSC and AML blasts but require association with ASCT (209, 210). However, targeting of the Lewis Y (LeY) antigen and NKG2DL CAR T-cells have also been proposed, but phase 1 trials have shown short response durations, despite less toxicity (211, 212). Combinations using several targets have been also proposed. Compound CAR T-cells targeting CD33 and CD123 are in development, and exhibit pronounced anti-leukemic activity (213). Nevertheless, CD123 and CCL-1 compound CAR T-cells might be useful for LSC targeting with limited hematopoietic toxicity (36, 177). Lastly, IL1RAP CAR T-cells have been described as having a potent effect on LSC in CML, and more recently in AML, without affecting healthy HSCs (214, 215). Interestingly this target is shared with AML blasts that overexpress IL1RAP and it is not only associated to IL-1 receptor but also to FLT3, KIT and other pro-inflammatory interleukin receptors (46). Lastly, integrative approaches using proteomics and transcriptomics could help to identify the better combinatorial CAR T-cells therapy. Some target pairwise combinations hold promise, such as CD33/CD70, CD33/ADGRE2 (CD312), CCL-1/CCR1 (CD191), and CCL-1/LILRB2 (CD85d) combinations (216), although clinical investigations are required to fully validate this approach.

Furthermore, microenvironment could play a major role in regulation of CAR T-cell functions and toxicities. Indeed, IL-15 may enhance anti-AML activity of CD123 CAR T-cells (217). Moreover, several costimulatory ligands are deregulated that induce T-cell inhibition (115). Thus, one proposed strategy was targeting inhibitory ligands expressed by AML in addition to LSC antigens targeting. Bispecific CD13-TIM-3 CAR T-cells reduced HSC toxicity (218), as well as a B7-H3 CAR T-cells (219). Besides, B7-H3 pan-cancer target was also studied in solid tumors (220). Preliminary reports show that PD-1 inhibitors also regulate CAR T-cell response, although few data are available (221). Furthermore, delivery of PD-1-blocking scFv CAR T-cells in pre-clinical investigations demonstrated interesting anti-tumor efficacy enhancement (222).

Lastly, several mechanisms were used to secure CAR T-cells administration. In order to avoid uncontrolled toxicity, the use of anti-CD52 alemtuzumab or CD20 protein co-expression in CD123 CAR T-cells for anti-CD20 rituximab targeting, is proposed (223). Integration to a suicide gene in the CAR construction, using inducible procaspase 9 by rimiducid, has been reported (224). A major side effect of CAR T-cell administration is cytokine release syndrome (CRS) resulting from the excessive production of those cytokines, particularly IL-6 (225). Similarly, immune effector cell-associated neurotoxicity syndrome (ICAN) is associated with excessive production of IL-1. Furthermore, the production of IL-1β, IL-6, and TNFα induces IL-10 and TGFβ productions that impair CAR T-cells functions (226). Anti-IL-1 (anakinra) and anti-IL-6 (tocilizumab) were described to counteract these two immune side effects (225). Of note, targeting IL1RAP might be an interesting manner to reduce pro-inflammatory side effect by reducing pro-inflammatory interleukin activation (194), but also by targeting monocytes that are assumed to produce IL-1 and IL-6 in CRS (214, 227). However, that suggestion needs to be demonstrated. Several challenges remain to be overcome as recently reported (228), and further investigations may provide a better understanding.

##### Transgenic T-Cell Receptor (TCR) T-Cells, Leading Competitors

Another adoptive T-cells transfer therapy that could be proposed is generated T-cells with a transgenic TCR specific to one antigen. A few tumor-specific proteins that act as leukemia-specific antigens (Tumor Specific Antigens, TSA), such as RUNX1-RUNX1T1, FLT3, and NPM1, have been described as being associated with AML (229), resulting from mutations (mutated TSA) or aberrant expression (aberrantly expressed TSA) from noncoding regions (230). C-terminal CLAVEEVSLRK sequence of NPM1c (ΔNPM1) binds and presents as HLA-A*02:01 (231). Anti-tumor activity by ΔNPM1-transgenic -cells receptor (tgTCR) T-cells against AML cells have been recently reported (232). Further investigations may yield interesting results. Subsequently *in vitro* T-cell recognition has been demonstrated in IDH1/2, FLT3-ITD and Ras-MAPK pathway mutations, and PML-RARα and DEK-CAN fusion proteins, that are considered as promising targets for CAR T-cells, tgTCR T-cells or a vaccine (233, 234). Other antigens such as WT1 that are considered as leukemia-associated antigens (Tumor-Associated Antigens; TAA) are not completely specific to AML cells. A phase 1 study of WT1-tgTCR T-cells highlights interesting survival rates that are greater than 12 months in patients with persistent tgTCR T-cells (235). Moreover, the use of allogeneic WT1-tgTCR T-cells following ASCT appear to prevent AML relapses (236). However, only HLA-A*24:02 presents WT1 peptides to T-cell receptors (TCR). Thus, WT1-transgenic TCR T-cells (tgTCR) are restricted to HLA-A*24:02 patients. Subsequently, further investigations are ongoing (229). WT1 is also a target investigated in vaccination studies, such as the multivalent WT1 peptide vaccine (237) or the dendritic cell vaccination (238). Myeloperoxidase- (MPO) targeting by MPO-tgTCR T-cells is also described as having potent anti-AML efficacy, but is restricted to HLA-B*07:02 presentation (239). Notably, resistance to MPO-tgTCR T-cells may appear *via* the downregulation of HLA. Hyaluronan-mediated motility receptor- (HMMR/Rhamm) tgTCR T-cells, which are similarly restricted to HLA-A*02:01 presentation, are reported as showing potent cytotoxicity toward not only AML cells, but also HSCs (240). In addition, overexpression of Melanoma Associated Antigen-A3 (MAGE-A3) in AML and MAGE-A3-targeting have been reported (241, 242).

Unlike antigens presented by the HLA major histocompatibility complex (MHC), minor histocompatibility antigens (MiHAs) are encoded by germline polymorphisms. In ASCT, differences between MiHA expression in malignant and normal hematopoietic cells of patients and donors, respectively, result in GVHD but also in graft-versus-leukemia (GVL), due to HLA and Killer-cell Immunoglobulin-like Receptor (KIR) mismatches (242, 243). This strategy is used in DLI, Cytokine-induced Killer cell (CIK) and donor NK cell infusions as well. But MiHA-targeting is limited by the low frequency (only 0.5%) of non-synonymous single nucleotide polymorphism (sn-SNP) generating HLA-associated peptides (244). MiHAs are being investigated in order to identify suitable targets in AML, such as the *via* the proteomic approach. The most common HLA haplotype presenting MiHAs in European Americans seems to be HLA-A*02:01; B*44:03 (245). Thus, MiHA-based immunotherapies should target antigens restricted to AML cells, well balanced in the population, but also demonstrating MHC binding and immunogenicity (242). In fact, the leukemia-associated minor H antigen 1- (HA-1) tgTCR T-cells, restricted to HLA-A*02:01, are used for the most advanced MiHA-based immunotherapy, regarding which further investigations are ongoing (NCT03326921) (246). Therefore, TSA-, TAA-, and MiHA-based therapies are mainly limited to their HLA restriction in addition to the small number of suitable targets (242). Lastly, to limit off-target toxicities induced by mispairing between the endogenous and the introduced TCR chains, a reported strategy is knocking-out the endogenous TCR by genome editing, as TALEN or CRISPR-Cas-9 approaches (247). This strategy is also investigated to generate universal engineered T-cells (198).

#### Immune Checkpoint Blockade and Microenvironment Targeted Therapies Enhance Immune Cell Therapies

As previously described, AML blasts present a deregulation of costimulatory ligand with concomitant changes in donor T-cells after ASCT. In addition, several inhibitory receptors are expressed by early differentiated memory and central memory T-cell in bone marrow of AML patients relapsing (248). Immune checkpoint blockade (ICB) antibodies have demonstrated efficacy in solid tumor with immune synapse restoration and tumor cell eradication (249). Then, several preclinical studies have investigated ICB in AML (36, 64). Superior efficacies of ICB nivolumab (anti-PD1), pembrolizumab (anti-PDL1), and ipilimumab (anti-CTLA4) in combination with azacytidine or other cytotoxic drugs have been reported in relapses following ASCT, or with other ICB (64). The use of Ipilimumab in AML relapse following ASCT has shown a CR rate of 23%, but use of ICB after ASCT needs particular caution regarding GVHD (250). Interestingly, response occurs in extramedullary disease. HMAs increase PD-1/PD-L1 expression in AML blasts (251). Moreover the azacytidine and nivolumab combination in R/R AML induced a CR/CRi rate of 22%, whereas the addition of ipilimumab improved the CR/CRi rate and median OS (252, 253). The consensus is that ICB may play an adjunct role to other interventions, and display limited efficiency when used alone (68, 254, 255). Multiple studies in search of further information are ongoing (65, 68, 256). TIM-3 and T Cell Immunoglobulin and Immunoreceptor Tyrosine-Based inhibitory motif domain (TIGIT) blockade are also being investigated. Moreover, ICB and CAR T-cells combination was proposed in solid tumor in the purpose to enhance antitumor activity, with promising results (257). That strategy could be also interesting to investigate in AML.

Furthermore, *in vivo* Treg depletion *via* immunotherapies based on bacterial toxins are also being investigated. A recent study reported preclinical results indicating that denileukin difitoxin, a diphtheria toxin, may target the IL-2 receptor and eliminate Tregs (68, 258). Another form of microenvironment targeting is achieved by the interruption of the CXCR4/CXCL12 signaling axis by CXCR4 monoclonal antibody, ulocuplumab, that showed interesting results in phase 1 trials with a CR/CRi rate of 51% (259). This blockade induces migration of HPCs and LSCs from the BM to peripheral blood. Several investigations in combination with LDAC or chemotherapy are ongoing (256). Further investigations may reveal novel and useful immunotherapies. In addition, several microenvironmental targets such as IL-1β pathway and IL-1 antagonists or β3-AR agonists for sympathetic neuropathy (such as mirabegron) (260), mitochondrial heterocellular transfer, and inhibition by tigecycline, fatty acid oxidation (FAO) and FAO inhibitor etomoxir that sensitizes AML cells to therapeutic challenge, NO synthase inhibitors (such as cavtratin) (261, 262) and endothelium activation (59), are under investigation.

## Global Strategies for AML Treatment

For patients eligible for intensive chemotherapy, the ELN 2017 panel recommends an induction therapy involving 3 days of treatment with an anthracycline, such as daunorubicin ≥60 mg/m², idarubicin 12 mg/m² or mitoxantrone 12 mg/m², combined with 7 days of continuous cytarabine infusion (100-200 mg/m²). Induction has to be followed by a consolidation therapy depending on ELN risk-stratification and age or comorbidities as follows: an intermediate dose of cytarabine (IDAC) (2-4 cycles of 1,000–1,500 mg/m² twice a day for of 3 days) for younger patients in favorable- and intermediate-risk groups (18–60/65 year-old) and IDAC (2–3 cycles of 500–1,000 mg/m² twice a day for 3 days) for older patients in the favorable-risk group (≥60/65 year-old) (2). No consolidation therapy has been shown to have an established value for intermediate- and adverse-risk older patients. Addition of midostaurin to intensive chemotherapy should be considered in case of FLT3-mutated AML (140). ASCT is recommended for intermediate- and adverse-risk younger patients, and for older patients with a low comorbidity index (2). These recommendations have been recently confirmed by the ESMO Guidelines Committee with a few updates (263). The benefits of prophylactic or pre-emptive DLI and maintenance with HMA, or other drugs, are still unclear and require further studies. Lastly, an oral HMA has reportedly improved OS during maintenance in ≥55 year-old patients who are in CR1 after intensive chemotherapy (264). Only tyrosine-kinase inhibitor based maintenance in BCR-ABL-positive AML following ASCT is recommended (263).

Salvage therapy consists of IDAC with or without anthracycline described as FLAG +/- IDA (fludarabine, cytarabine, idarubicin, and additional granulocyte-stem cells factor). Other salvage regimens, such as mitoxantrone, etoposide and cytarabine (MEC), have shown overlapping results (2). The sequential transplant conditioning regimen has also been proposed as FLAMSA-RIC (cytarabine/amsacrine salvage regimen and fludarabine based RIC regimen) when either a donor or the donor source (MUD, haploidentical, or UCB) is immediately available (263). Patients ineligible for intensive chemotherapy as a first line may benefit from HMA (azacytidine or decitabine). The challenging options for such frail patients are low, sub-cutaneous doses of cytarabine (LDAC) or best supportive care.

For adults, new drugs have been recently approved by the FDA and EMA as follows: CPX-351 for AML with MRC-AML or tAML, gemtuzumab ozogamicin in addition to standard induction chemotherapy for CD33-positive AML with favorable- and intermediate-risk, gilteritinib for R/R FLT3-mutated AML and glasdegib in combination with LDAC for newly-diagnosed AML cases who are ≥75 year-old, or have comorbidities. In addition, the FDA has also approved ivosidenib for ≥75 year-old ineligible patients with IDH1-mutated AML or R/R IDH1-mutated AML, enasidenib for R/R IDH2-mutated AML, and venetoclax in combination with azacytidine, decitabine, or LDAC for newly diagnosed ≥75 year-old AML patients who are ineligible for standard intensive chemotherapy. Nevertheless, as discussed by Estey et al., several concerns have been reported as problematic issues confronting the assessment of fitness for standard induction therapy, such as the intensity of compared induction, problematic endpoints and approvals for unstudied populations (265). Fitness evaluation *via* a standardized score, which is similar to that for ASCT or geriatric assessment, should be performed (266, 267). Moreover, the OS rate and quality of life assessments may be more useful for patients.

Recently, DiNardo et al., proposed that these new therapies should be discussed in detail (268). Personalized medicine may be offered to many patients depending on fitness or phenotypic and molecular disease characteristics. Development of targeting drugs such molecular inhibitors or mAb may be useful as a third drug in intensive induction regimens, in salvage therapy or in maintenance, depending on specific mutations or cell surface markers. ICB and venetoclax appear to show potential for salvage therapy in association with other drugs, but further studies are required. Concerning maintenance therapy following ASCT, despite the identification of driver mutations and development of inhibitors, strategies adapted to residual disease, monitored by MRD assessment after ASCT, need further investigation, before specific therapies can be proposed at the appropriate moment (269, 270). Azacytidine and DLI are the most widely used therapies following ASCT, in addition to immunosuppressive drug adaptation (111, 168). In fact, for patients undergoing FLT3 inhibitor treatment prior to ASCT the only therapy the EBMT Acute Leukemia Working Party of recommends is ASCT consolidation and FTL3 inhibitor maintenance for at least 2 years following ASCT (146).

**Figure 3:**
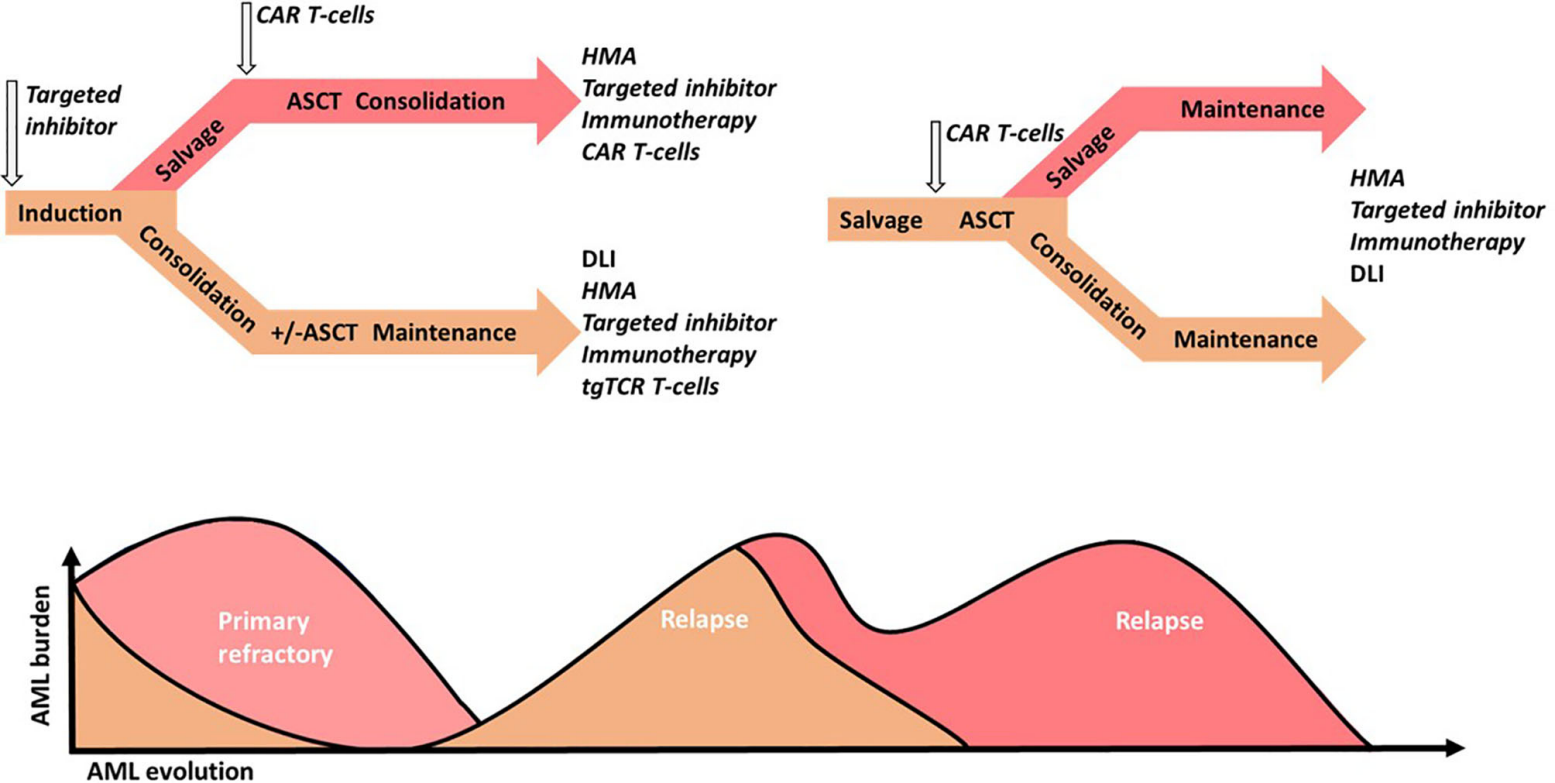
Acute Myeloid Leukemia (AML) intensive strategy perspectives in younger patients. Standard treatments are in regular small captions, putative investigated treatments are in italics. Standard chemotherapy could be combined with targeted drug inhibitors (targeted inhibitor), such as FLT3 inhibitors or targeted chemotherapeutic agents such as CD33-conjugated antibodies. In the case of first complete response (CR1), intermediate and high-risk patients with or without maintenance therapy are subjected to consolidation via chemotherapy, with or without targeted drug inhibitors, such FLT3 inhibitors, followed by allogeneic hematopoietic stem cell transplantation (ASCT). In the case of primary refractory AML, salvage therapy may be improved via chemotherapy by the addition of a hypomethylated agent (HMA), targeted inhibitors, targeted-drug immunotherapy (immunotherapy) or chimeric antigen receptor (CAR) T-cells, followed by ASCT. Measurable residual disease or low burden relapse may be treated with HMA or a donor lymphocyte infusion (DLI) in case of ASCT, and improved through the addition or the single use of a target inhibitor, immunotherapy or transgenic T-cell receptor (tgTCR) T-cells. Relapses with greater AML burden may be treated via chemotherapy and improved via the addition or single use of a targeted inhibitor, immunotherapy, or CAR T-cells. Next, ASCT following chemotherapy, single or several targeted therapy regimens depending on the response or CAR T-cells may be performed. Consolidation with or without maintenance may also be performed using HMA, donor lymphocyte infusion (DLI) in case of ASCT, or targeted therapy.

Finally, no leukemia-specific surface marker common to all patient exists. A major concern in CAR T-cells therapy, where the targeted cells share some phenotypic features with HSCs, is myeloablative activity of CAR-T cells *via* HSC targeting. The dilemma is reducing the potency of CAR T-cells and may increase the risk of disease escape (202). CAR T-cells may be considered as a bridge-to-transplant therapy in the face of significant or pan-myeloid cell activity. Indeed, ASCT is proposed in refractory AML but with a poorer outcome due to AML burden (2). Thus, CAR T-cells therapy may reduce the AML burden and act as an interesting salvage therapy that induces a greater response rate prior to ASCT. Moreover, MRD negative status before ASCT is associated with greater PFS and OS (271). Therefore, MRD monitoring may guide treatment intensification strategies before ASCT and CAR T-cells use, as well. While developing new targets for CAR T-cells with potent smaller hematopoietic toxicity, an ASCT should be prepared for salvage therapy in case CAR T-cells fail or hematopoietic toxicity is induced. Thus, CAR T-cells are more appropriate for younger and fitter patients. Better assessment and understanding of adverse toxicity risk factors and toxicity management may permit the use of CAR T-cells in R/R AML, high risk AML or older patients. In addition, the use of MiHA-, TSA-, and TAA-based immunotherapies, such as tgTCR, T-cells or vaccination approaches, appear to be more potent in the context of low burden disease (242). Thus, MRD monitoring studies may provide guidance for earlier use of T-cell therapies (**Figure 3**).

## Data Availability Statement

The original contributions presented in the study are included in the article/supplementary material. Further inquiries can be directed to the corresponding author.

## Author Contributions 

XR, MD, and CF designed the study. XR collected data, wrote the draft manuscript and drew the figures. MN improved the figures. All authors contributed to the article and approved the submitted version.

## Funding

Association « Nausicaa combat sa Leucémie » (non profit association 1901); Gift 2017-03, 2018 and 2019. SFGMTC/Association Capucine Grant 2013-AO2. Supported by the MiMedi project funded by BPI France (no. DOS0060162/00) and the EU through the European Regional Development Fund of the Region BFC (no. FC0013440). French Blood Center (EFS, DRVI) grants 2018 and 2019.

## Conflict of Interest

The authors declare that the research was conducted in the absence of any commercial or financial relationships that could be construed as a potential conflict of interest.
